# Heritability of Stroop and flanker performance in 12-year old children

**DOI:** 10.1186/1471-2202-5-49

**Published:** 2004-12-03

**Authors:** John F Stins, G Caroline M van Baal, Tinca JC Polderman, Frank C Verhulst, Dorret I Boomsma

**Affiliations:** 1 Department of Biological Psychology, Free University of Amsterdam, Van der Boechorststraat 1, 1081 BT Amsterdam, the Netherlands; 2 Department of Child and Adolescent Psychiatry, ErasmusMC-Sophia Children's Hospital, PO Box 2060, 3000 CB Rotterdam, The Netherlands

## Abstract

**Background:**

There is great interest in appropriate phenotypes that serve as indicator of genetically transmitted frontal (dys)function, such as ADHD. Here we investigate the ability to deal with response conflict, and we ask to what extent performance variation on response interference tasks is caused by genetic variation. We tested a large sample of 12-year old monozygotic and dizygotic twins on two well-known and closely related response interference tasks; the color Stroop task and the Eriksen flanker task. Using structural equation modelling we assessed the heritability of several performance indices derived from those tasks.

**Results:**

In the Stroop task we found high heritabilities of overall reaction time and – more important – Stroop interference (*h*^2 ^= nearly 50 %). In contrast, we found little evidence of heritability on flanker performance. For both tasks no effects of sex on performance variation were found.

**Conclusions:**

These results suggest that normal variation in Stroop performance is influenced by underlying genetic variation. Given that Stroop performance is often hampered not only in people suffering from frontal dysfunction, but also in their unaffected relatives, we conclude that this variable may constitute a suitable endophenotype for future genetic studies. We discuss several reasons for the absence of genetic effects on the flanker task.

## Background

The Stroop test [1] is arguably the best-known neuropsychological test to tap attentional (dys)function. In the color words version of this test the instruction is to attend to the color of the ink in which a word is printed and name this color aloud. At the same time, the printed words may also read certain color names that are different from the color of the ink in which it is printed. As has been observed on numerous occasions, there is a strong tendency to respond to the content of the word, and not to the ink color. This is evidenced by an increase in response time and a decrease in accuracy relative to a neutral control condition.

The Stroop test has been used both to tap fundamentals of human information processing (e.g. [2]), and as a clinical aid to assess attentional dysfunction, e.g., due to a frontal or fronto/parietal deficit. Brain imaging and neurological studies consistently point to the prefrontal cortex (PFC) as the site involved in resolving the response conflict. As a consequence, people suffering from attentional impairments, caused by prefrontal abnormalities (developmental or acquired), tend to suffer more from Stroop interference than controls. For example, the test succesfully differentiates unaffected controls from people suffering from schizophrenia (e.g., [3]). In a similar vein, people suffering from attention-deficit/hyperactivity disorder (ADHD) suffer from Stroop interference ([4]; see also [5]), although a recent meta-analysis cast some doubt about the usefulness of the Stroop task in differentiating people with ADHD from controls [6].

There now exist numerous versions of the Stroop test. For example, instead of using color words, researchers have adopted more ecologically relevant items, such as emotion words, pictures of food items or of threatening objects, etc. In addition, it is now also common to use computerized versions of the Stroop task, permitting a trial-by-trial analysis of performance. But what all these different Stroop versions have in common is that the subject is always presented with a stimulus that simultaneously activates two conflicting response channels; one response is activated by the instructions, whereas the other response is activated by elements in the array that strongly invite an alternative – yet incorrect – response. In order to resolve this response conflict the subject has to direct attention to task relevant information and ignore information from the task irrelevant channel. The time needed to resolve this conflict is derived using subtractive logic, and can be used as an index of the efficiency of the attentional system under investigation.

A task that is less widely used in clinical circles, but that also indexes the efficiency of the frontal network is the Eriksen flanker task. In the arrow version of this task, subjects have to respond to the direction of a left or right pointing arrow, and ignore flanking arrows that point in the opposite direction as the target arrow [7]. Similar to the Stroop task, there is a tendency to respond to the distracting flanker elements, and subjects have to resolve this response conflict prior to emitting the designated response. It is consistently found that response times are elevated due to the target-flanker incongruity, relative to a neutral control condition where target and flankers are congruent (that is, they all point in the same direction). There is evidence that the Stroop task and the flanker task are supported by the same cognitive system. For example, using functional magnetic resonance imaging (fMRI) it was found that both tasks activated largely overlapping brain regions, viz. the anterior cingulate cortex (ACC) and the left prefrontal cortex [8]. In addition, and similar to the Stroop task, subjects with ADHD spend more time resolving the conflict between the competing responses than controls (e.g., [9]). Also, adult subjects with ADHD showed consistent underactivation in the ACC (cognitive division) during a counting version of the Stroop task, compared to controls [10].

The Stroop task (and, to a lesser extent, the flanker task) has thus acquired a strong neuropsychological validation, and is nowadays widely used in clinical settings. However, studies adopting an individual differences paradigm have revealed that the time needed to resolve the response conflict in the Stroop task does not predict the time needed to resolve the response conflict in the flanker task. In an earlier study we [11] found that the interference scores between the tasks were uncorrelated. A similar finding was reported by [12] using slightly different task versions.

Further insight into the nature of these interference tasks might be gained by adopting a genetic perspective on normal and abnormal frontal functioning. A wealth of studies has now shown that many frontal psychopathologies are influenced by genes. For example, the heritability of ADHD is estimated to be around 80% (e.g.[13]). In a similar vein, the heritability of attention problems as established by questionnaires is estimated to be around 70 – 90% (e.g. [14]). However, genetic studies are often hampered by the fact that psychopathologies are multifacetted and complex. A recent line of inquiry has started to use a 'bottom-up' approach, trying to decompose the complex phenotype (behavior) into a set of variables that are thought to represent more basic processes or traits. In this so-called endophenotypic approach the search is for neuro-behavioral vulnerability markers that are somewhere intermediate the genes and the disease [15,16]. Endophenotypic measures gathered in children can be used to assess genetic vulnerability to adult psychiatric disorders [17]. In this paper we will try to assess whether, and to what extent Stroop and/or flanker performance can qualify as genetic indicators for frontal abnormalities. The usesfulness of measures of Stroop performance in a genetically informative design has recently been demonstrated in a study [18] that compared Stroop performance among children suffering from ADHD, their unaffected sibs, and a group of controls. It was found that not only the children with ADHD, but also their unaffected sibs suffered more from Stroop interference than the controls. In a similar vein, it was found that not only euthymic bipolar and schizophrenic patients, but also their unaffected first-degree relatives suffered from increased Stroop interference, relative to a healthy control group [19,20]. However, another study [21] failed to find deteriorated Stroop performance in unaffected ADHD sibs.

The usefulness of measures of flanker performance in a genetically informative design was demonstrated by a series of studies conducted by Fan and co-workers. Using a sample of healthy monozygotic (MZ) and dizygotic (DZ) twins, it was tested whether genetic variation contributed to variations in performance on basic attentional tasks [22]. These tasks were designed to tap distinct attentional brain networks (see also [23]). Of interest is performance on the flanker task, which was supposed to index the efficiency of the dopamine rich frontal executive network. Performance on this task indeed showed evidence of heritability. In a follow-up study, 200 subjects were genotyped, and were tested on a range of attention tasks. Modest associations were then found between genetic polymorphisms of several genes implicated in frontal (dys)function, such as *drd4 *and *dat1*, and the efficiency of the frontal executive attention network [24]. Using the same twin methodology, it was also found [25] that performance on the flanker task was heritable. In addition, there was a correlation between flanker performance and IQ, and this correlation was completely mediated by a common set of genes.

In this paper we ask whether variation in *normal *Stroop and flanker performance is caused by genetic variation. By using monozygotic twins, who share all their genetic material, and dizygotic twins, who share on average half of their segregating genes, the influence of genetic factors and environmental factors can be teased apart. If genetic effects are important, then members of monozygotic twin pairs will be more similar than members of dizygotic twin pairs in test performance. Conversely, if MZ twins show the same degree of resemblance as DZ twins, influences of environmental factors that are shared by both twins (e.g., the school or family environment) will be important. The contributions of additive genetic factors, shared environmental factors and unique environmental factors for explaining the variance observed for these measures can be explored using this twin design. If Stroop and/or flanker performance is found to be heritable, we will have a further genetic (in addition to neuropsychological) validation of these tasks with respect to frontal dysfunction. Furthermore, a high heritability of performance measures may ultimately help to unravel the genetic pathways of complex psychiatric traits.

This paper is a follow-up to a previous paper, where we reported behavioral data on Stroop and flanker performance [11]. The current paper extends the previous one by investigating genetic effects on variation in performance.

## Results

For the Stroop task, the data from 5 subjects (3 first born twins and 2 second born twins) could not be analyzed because they failed to comply with the instructions. Visual inspection of the data revealed that a few subjects had an extremely high Stroop interference score. Four subjects whose interference score was larger than 120 s were excluded from the analysis.

Due to technical problems the data of 24 subjects for the flanker task were not stored or collected. Furthermore, there were 2 subjects who had an extremely high error score (> 20 errors out of 80 trials). These subjects were excluded from the analyses.

### Descriptives

Table 1 shows the time to complete each of the three cards, separate for the first-born twins and the second born twins. The table reveals a clear increase in performance time from Card 1 to Card 2 to Card 3. The analysis of variance (ANOVA) for the first-born twins showed that there was a significant effect of card type, F(2, 278) = 1351.7, p < .001. The main effect of sex was not significant (p > .1), nor its interaction with card type. For the second born twins a near-identical pattern of results was found: a main effect of card type, F(2, 276) = 1236.2, p < .001, and no effects involving sex. Thus, we obtained a robust Stroop effect, and this was not affected by the sex of the subject. Accuracy data can be found in Table 2.

For the flanker task we found the following effects: For the first born twins the main effect of stimulus type was significant, F(1, 128) = 463.00, p < .001. Congruent stimuli yielded faster RTs than incongruent ones (556 vs. 662 ms). The main effect of sex was not significant (p > .1), nor its interaction with stimulus type. For the second born twin the main effect of stimulus type was significant, F(1, 132) = 556.02, p < .001. Again, congruent stimuli yielded faster RTs than incongruent ones (551 vs. 653 ms). Also, the main effect of sex was significant, F(1, 132) = 5.38, p < .05. Boys were somewhat faster than girls (587 vs. 618 ms).

The same analyses done on the error rates yielded a comparable pattern of results. For the first born twins the main effect of stimulus type was significant, F(1, 128) = 84.86, p < .001. Congruent stimuli yielded more correct responses than incongruent ones (98.1% vs. 94.9%). In addition, the main effect of sex was significant, F (1, 128) = 4.57, p < .05, as was its interaction with stimulus type, F (1, 128) = 5.03, p < .05. These effects indicate that boys tend to respond somewhat less accurate than girls on incongruent trials. For the second born twins also the main effect of stimulus type was significant, F(1, 132) = 115.18, p < .001. Again, congruent stimuli yielded more correct responses than incongruent ones (98.8% vs. 93.9%). The main effect of sex was not significant, but the interaction was significant, F(1, 132) = 4.05, p < .05. Again, boys tended to respond somewhat less accurate than girls on incongruent trials.

In order to test whether for the flanker task there was a trade-off between response speed and accuracy, we simply correlated RTs with accuracy, separately for the first born and second born twins. A possible speed-accuracy trade-off would manifest itself as a significant negative correlation between mean reaction time and percentage of errors. For the first born twins we found a significant positive correlation (r = .37, p < .001). But this correlation appeared to be due to a handful of subjects who were both quite slow and error prone. For the second born twins the correlation was small (r < .1), and not significant. Thus, we conclude that in our sample there was no evidence of a speed-accuracy trade-off.

A similar analysis was done for the Stroop task. We correlated the average completion time of Card 3 with the number of errors commited with Card 3. We did not correlate speed with the number of corrections, because these measures are not independent. For both the first born twins and the second born twins we found a significant positive correlation (r = .31, and r = .21, respectively. p's < .05). Thus, subjects who were slow also tended to be inaccurate. However, the distribution of the number of errors was rather skewed (most subjects made 0 or 1 errors), which makes it difficult to interpret these correlations. So, similar to the flanker task, we conclude that there was no evidence of a speed-accuracy trade-off.

### Genetic analyses

Table 3 shows twin correlations of times to complete Card 1, 2 and 3, and of the interference effect (Stroop effect; difference between Card 3 and Card 2). For the 3 cards, a very consistent pattern is seen: MZ correlations are high, around .7, and DZ correlations are approximately half, implying the existence of genetic influences and unique influences, with a heritability of around 70%. The twin correlations of the interference effect are somewhat lower, probably because difference scores tend to have a lower reliability[11]. MZ correlations were around .5 and DZ correlations were lower, pointing to a heritability of about 50%.

Table 4 shows the twin correlations for the overall RT and the flanker interference effect (RT [incongruent] *minus *RT [congruent]). The pattern of twin correlations is hardly indicative of genetic effects on performance. Even though the MZ twin correlations on response speed were higher than the DZ correlations, the highest twin correlation was obtained with the DOS zygosity group. Furthermore, the highest twin correlation for the flanker effect was obtained with the DZF group. In addition, the twin correlations within the monozygotic groups were low. We therefore conclude that there were no genetic effects of flanker performance.

The twin correlations obtained with the Stroop task thus appeared strongly indicative of genetic effects. Using structural equation modelling, these effects were formally tested. But prior to testing we had to establish whether there were significant differences in variances across sex and zygosity, since one of the assumptions underlying structural equation modelling is the assumption of homogeneity of variances. We conducted the Levene test on all variables, separately for the first-born and the second born twins. For none of the variables the Levene test yielded a significant effect, with the possible exception of the completion time of Card 1 for the second born twins, F(3, 136) = 2.664, p = 0.05. So, we felt it was legitimate to use structural equation modelling to test for genetic effects.

Additional file 1 shows the results. The full ACE model, which allowed for sex differences in parameter estimates fitted well to the data (χ^2 ^ranged from 7.732 to 8.883, df = 9, p's ranged from .448 to .561), with the possible exception of Card 1 (χ^2 ^= 15.339, df = 9, p = 0.082), although the most parsimonious model for Card 1 fitted slightly better (χ^2 ^= 17.240, df = 13, p = 0.189). Sex differences in parameter estimates could be discarded from the models, although they were almost significant for Card 3 (χ^2 ^= 7.652, df = 3, p = 0.054). Common environmental influences were not necessary to describe the data, but additive genetic influences explained a significant part of the variance in all 4 variables. Heritabilities for the 3 cards were 75%, 70% and 74% respectively, with confidence intervals indicating that these were well above half the variance. Heritability of the interference effect was 49%, with a 95% confidence interval between 29 and 64%.

## Discussion

In this study we assessed the heritability of performance on two well-known response interference tasks: the color word Stroop task and the Eriksen flanker task, using a large sample of 12-year old twins. The aim was to test whether Stroop performance and/or flanker performance could qualify as a suitable endophenotype for genetic frontal abnormalities, such as ADHD. First, we found that the time to complete each of the three cards was highly heritable. This may represent a general factor related to processing speed and/or rapid naming speed. Of greater importance was the finding that the interference score (the difference between completion times of Card 2 and 3) was also heritable: nearly 50% of the variation in performance was due to genetic variation. Thus, the efficiency of the network that deals with response conflict is -in part- under genetic influence.

For the theoretically similar flanker task, however, there was little evidence of genetic influences on performance. Even though the MZ twin correlations on response speed were higher than the DZ correlations (as in [25]), the highest correlation was observed for the DOS twins, for which we have no explanation. In addition, there was no evidence of genetic influences on variations in the size of the flanker effect. Variation in performance thus simply appeared to be due to noise. This latter finding is at odds with a previous study where it was found that variation in the size of the flanker interference effect was 89% due to variations in genes [22]. This discrepancy could of course be due to minor differences between task versions. For example, the flanker test adopted by [22] was embedded in a visual orienting paradigm. But it could also be the case that genetic effects on flanker performance are somehow age specific. Our age group was 12 years old, whereas Fan et al.'s [22] age group was between 14 and 42 years of age. It is well known that heritabilities of different traits vary with age. For example, the heritability of IQ is known to steadily increase with increasing age (e.g. [26]), and it could be the case that genetic effects on flanker performance only emerge at a later age. Finally, it could be that Fan et al. [22] have obtained a false positive result, due to their somewhat modest sample size (26 MZ pairs and 26 DZ pairs).

The question now is whether Stroop and flanker performance can qualify as a suitable endophenotype of frontal pathologies. Recently a list of 5 criteria was compiled that are ideally possessed by endophenotypes [15]. Criteria 3 to 5 deal with the relationship between phenotype and endophenotype. In brief, there should be a high correlation between the phenotype and endophenotype, this correlation should be based in genetics, and the correlation should be theoretically meaningful. In our Introduction we have briefly touched upon the relationship between performance on response interference tasks and high-level phenotypes, such as the efficiency of the frontal executive network. Our selective review of the literature indicated that there was a clear genetic link between Stroop performance and frontal pathologies, whereas evidence for a the genetic link between flanker performance and frontal pathologies was less conclusive.

Criteria 1 and 2 of [15] state that the endophenotype should be reliable and heritable. With respect to heritability, we have demonstrated that – at least for this age group – there is strong evidence for genetic influences on Stroop performance, but not on flanker performance. With respect to reliability, we have no test-retest data but we can assess reliability by examining the MZ correlations, because these correlations provide a lower limit to reliability [27]. Inspection of Table 2 reveals that the Stroop performance measures are characterized by high MZ correlations, which implies high reliabilities. The flanker performance measures reported in Table 3, however, revealed quite low MZ correlations. This finding, in combination with a low split-half reliability (reported in [11]) leads us to conclude that Stroop performance provides a more reliable measure than flanker performance.

## Conclusions

We have found evidence for the existence of strong genetic effects on conflict resolution, although the effects are task dependent. We conclude that performance on the Stroop test yields a better endophenotype for frontal (dys)function than performance on the flanker task. So, despite the overlapping regions of brain activation in the Stroop and flanker tasks, and despite their face-value similarity, we believe that these interference tasks differ in important, yet unknown ways.

## Methods

### Subjects

The subject group consisted of a group of 290 12-year old twins. There were 33 monozygotic male pairs (MZM), 24 dizygotic male pairs (DZM), 45 monozygotic female pairs (MZF), 16 dizygotic female pairs (DZF), and 27 opposite sex pairs (DOS). The twins participate in a longitudinal study of attention and attention problems. The twins are registered in the Netherlands Twin Registry (NTR), which is hosted by the Vrije Universiteit of Amsterdam [28]. The twins were randomly selected from the NTR subject pool. None of the children suffered from severe mental or physical impairments.

Twin pairs were first asked in writing whether they were willing to participate in the study. Permission was also asked of the parents or caretakers. If permission was granted, the families received further information on the study, and were invited to come to the campus site to do the tests. The study was approved by the local Ethics Committee, and on the day of testing the children and their parents / legal representatives signed an informed consent form.

### Procedure

Twins were tested on the same day. The children performed a range of neuropsychological tests that lasted approximately 4 hours per child. All tests were performed in the same order. Here we focus on the Stroop Color and Word Test [1] and the Eriksen flanker test. In the Stroop test, subjects complete 3 cards, each with 10 columns of 10 items. Subjects have to name aloud the items on each card, from the top-left corner to the bottom-right corner. Card 1 involves naming the words 'red', 'green', 'yellow' and 'blue' printed in black ink. Card 2 involves naming the colors of squares that are printed in different colors. Card 3 involves naming the ink color that the words 'red', 'green', 'yellow' and 'blue' are printed in. In Card 3 word content and ink color never match, i.e., all color words are incongruent. Speed and accuracy are stressed in the instructions. Each card is scored as the time (in seconds) to complete the card. Time is recorded by the experimenter using a stopwatch. The experimenter also recorded the number of errors (the wrong item is named, or an item is skipped), and the number of corrections (the wrong item is named, but the subject immediately corrects himself afterwards). Note that in this task we do not have trial-by-trial information on speed and accuracy; we only have summary scores across the entire Stroop card.

In the Eriksen flanker task subjects were presented with a horizontal array of 5 arrows. Subjects were instructed to attend to the direction of the center arrow, and ignore the 4 flanking ones. Subjects had to press the left key to a left facing center arrow, and the right key to a right facing center arrow. The flanking arrows could either all point in the same direction as the target arrow (e.g., < < < < <; congruent condition), or they all pointed in the opposite direction (e.g., < < > < <; incongruent condition). Subjects received 40 congruent and 40 incongruent trials in a random order. For each trial, the computer stored the RT and whether the correct key was pressed. The number of valid cases in the flanker task is lower than in the Stroop task. This was due either to practical problems (some data were not collected or not stored on the computer), or because some subjects made an extremely high number of errors (less than 75 % correct). Further details of the Stroop task and the flanker task can be found in [11].

### Data analysis: test of means

In order to test for the effects of card type and sex on Stroop performance we performed an analysis of variance on the completion times with card type (1, 2, and 3) as within-subjects factor, and sex (males and females) as between subjects factor. For the flanker task, we performed an ANOVA on the mean correct response times and on the mean percentages correct, with stimulus type (congruent vs. incongruent) as within-subjects factor, and sex (males and females) as between subjects factor. The same analysis was done for the error rates. These analyses were done separately for the first-born twins and the second born twins, because twins within a family do not yield independent data. We adopted an alpha-level of .05.

From the completion times we also calculated the size of the Stroop effect (i.e., the interference score), which is simply defined as Completion time Card 3 *minus *Completion time Card 2. There exists another method to determine the size of Stroop effect, which also takes into account the completion times of Card 1 [6,29]. But a preliminary analysis revealed that this method and the method used by us yielded virtually identical results, so we present no data based on the method proposed by [29].

### Data analysis: genetic analysis

Data from monozygotic (MZ) and dizygotic (DZ) twins were used to decompose the variance in performance on the both tasks into a contribution of the additive effects of genes, environmental influences that are shared by twins living in the same family, and environmental influences that are not shared by twins. Resemblance between MZ twins is an effect of both their common genetic constitution and their shared environment. Because DZ twins share on average half of their segregating genes, the shared environment contributes fully, but genetic factors only partly to their resemblance. Therefore, if the degree of MZ resemblance on some measure is higher than the degree of DZ resemblance we have strong evidence for the influence of genetic effects.

Pearson correlations were calculated for the different measures between first born and second born twins for all zygosity groups. A first indication of the heritability can be derived by doubling the difference between correlations for MZ twins and those for DZ twins [*h*^2 ^= 2(r_MZ_-r_DZ_)] [27].

**Figure 1:**
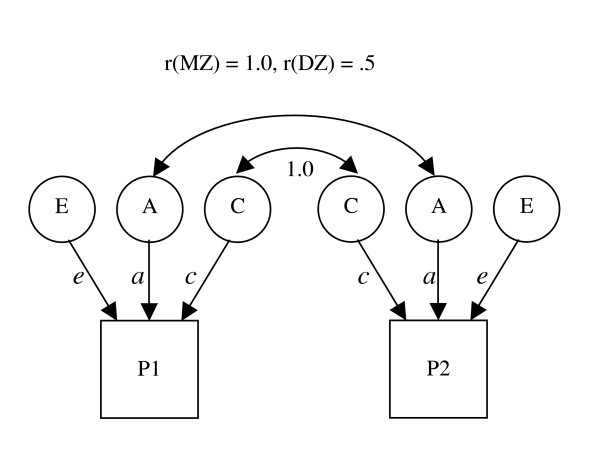
**A path diagram of a univariate ACE model **A path diagram of a univariate ACE model (A = Additive genetic factors, C = shared of common environmental factors, E = nonshared environmental effects) in which the three latent independent variables influence variation (indicated by single headed arrows) in a particular behavior or phenotype (P; P1 for twin 1 and P2 for twin 2). MZ = monozygotic; DZ = dizygotic. Partial regression coefficients (letters *a*, *c *and *e*) reflect the degree of relationship between the latent variables and the phenotype. Double headed arrows indicate the correlations among variables.

A structural equation modeling approach as implemented in Mx [30] was used for genetic data analysis. The dependent variables were analyzed using a model including three latent independent factors – additive genetic factors (A), shared or common environmental factors (C) and non shared or unique environmental factors (E) – that influence variation in a particular phenotypic measure of attention (P). A path diagram of an ACE model is presented in Figure 1. Because these latent factors are standardized to have a variance of 1.0, the double-headed arrow connecting them represents the correlation among them. The correlation between genetic effects in twin 1 and twin 2 is 1.0 for MZ twins and 0.5 for DZ twins. These between-twin correlations are represented as fixed parameters in the Mx model, as is the correlation between the common environmental factors (shared by both twins of a twin pair), which is fixed to unity for both twin groups. Parameters *a*, *c *and *e *represent the influence of genes, common environment and unique environment on the phenotypes (P) of twin 1 and twin 2. The total variance of the phenotype (P) = *a*^2 ^+ *c*^2 ^+ *e*^2^. The heritability (*h*^2^) is calculated as *a*^2^/V_P_.

To test if parameter estimates are equal for boys and girls the fit of a model with constrained parameter estimates for *a*, *c *and *e *to be equal across sexes was compared to one in which they were allowed to vary. After this, the significance of c and a was investigated by dropping them one by one from the model and comparing the fit of a full model to that of a reduced model. The chi-squared statistic is computed as twice the difference between the likelihood for the full model (-LL_0_) and that for a reduced or constrained model (-LL_1_) (χ^2 ^= 2 × (LL_0_-LL_1_)) and is tested against the difference in degrees of freedom between the two models.

## Authors' contributions

JFS drafted the manuscript, and performed the descriptive analyses. GCMvB performed the genetic analyses. TJCP collected the data. FCV and DIB conceived of the study, and participated in its design and coordination. All authors read and approved the final manuscript.

## Supplementary Material

Additional File 1 Model fitting and parameter estimates Model fitting and parameter estimates of heritability (*h*^2^), common environmentability (*c*^2^), and unique environmentability (*e*^2^), for completion times of the three cards, and of the Stroop interference score. Best fitting models are shown in bold. Parameter estimates are given in percentages (95% confidence interval in brackets). Click here for file

## Figures and Tables

**Table 1:** Completion time for each of the three Stroop cards in seconds (standard deviations in brackets), separate for first and second born twins (sample size is given in brackets).

*Card type*	*first born twins (141)*	*second born twins (140)*
Card 1	52.0 (8.2)	51.0 (7.7)
Card 2	71.0 (11.8)	69.1 (11.3)
Card 3	117.5 (22.9)	117.2 (24.6)
Interference score (3 *minus *2)	46.5 (16.6)	48.2 (17.3)

**Table 2:** Accuracy data for each of the three Stroop cards, separate for first and second born twins (sample size is given in brackets).

*Card type*	*Number of errors, first born twins (136)*	*Number of errors, second born twins (135)*	*Number of corrections, first born twins (136)*	*Number of corrections, second born twins (135)*
Card 1	.19	.19	.24	.27
Card 2	.61	.51	1.42	1.74
Card 3	1.32	1.44	3.15	3.36

Shown are the mean number of errors (the wrong item is named, or an item is skipped), and the number of corrections (the wrong item is named, but the subject immediately corrects himself afterwards). Note: the sample size is somewhat smaller than for the completion times because accuracy was not recorded with some subjects.

**Table 3:** Twin correlations for the completion times of each of the three cards and the interference score (95% confidence interval in brackets).

Zygosity	N Twin1	N Twin2	Card 1	Card 2	Card 3	Interference score (3 *minus *2)
MZM	32	33	0.68 (0.45 – 0.83)	0.78 (0.60 – 0.88)	0.75 (0.55 – 0.87)	0.44 (0.12 – 0.68)
DZM	23	22	0.41 (0.02 – 0.69)	0.47 (0.08 – 0.74)	0.37 (-0.03 – 0.67)	0.11 (-0.30 – 0.49)
MZF	43	43	0.70 (0.51 – 0.82)	0.60 (0.36 – 0.76)	0.70 (0.51 – 0.83)	0.55 (0.29 – 0.73)
DZF	16	16	0.25 (-0.24 – 0.64)	0.38 (-0.10 – 0.72)	0.68 (0.32 – 0.87)	0.32 (-0.17 – 0.68)
DOS	27	26	0.29 (-0.08 – 0.60)	0.31 (-0.07 – 0.61)	0.19 (-0.18 – 0.52)	0.10 (-0.28 – 0.46)

Note: MZM = monozygotic males; DZM = dizygotic males; MFZ = monozygotic females; DZF = dizygotic females; DOS = opposite sex pairs. N Twin 1 is the number of first born twins per zygosity group; N Twin 2 is the number of second born twins.

**Table 4:** Twin correlations for the RTs on the Eriksen flanker task, and the flanker interference score (flanker effect).

Zygosity	N Twin1	N Twin2	Overall RT	Flanker effect
MZM	29	31	0.38	0.12
DZM	20	19	-0.01	-0.07
MZF	41	41	0.35	0.09
DZF	14	15	0.18	0.52
DOS	25	26	0.55	0.04

Note: MZM = monozygotic males; DZM = dizygotic males; MFZ = monozygotic females; DZF = dizygotic females; DOS = opposite sex pairs. N Twin 1 is the number of first born twins per zygosity group; N Twin 2 is the number of second born twins.
